# Learning Latent Variable Gaussian Graphical Model for Biomolecular Network with Low Sample Complexity

**DOI:** 10.1155/2016/2078214

**Published:** 2016-10-23

**Authors:** Yanbo Wang, Quan Liu, Bo Yuan

**Affiliations:** Department of Computer Science and Engineering, Shanghai Jiao Tong University, Shanghai 200240, China

## Abstract

Learning a Gaussian graphical model with latent variables is ill posed when there is insufficient sample complexity, thus having to be appropriately regularized. A common choice is convex *ℓ*_1_ plus nuclear norm to regularize the searching process. However, the best estimator performance is not always achieved with these additive convex regularizations, especially when the sample complexity is low. In this paper, we consider a concave additive regularization which does not require the strong irrepresentable condition. We use concave regularization to correct the intrinsic estimation biases from Lasso and nuclear penalty as well. We establish the proximity operators for our concave regularizations, respectively, which induces sparsity and low rankness. In addition, we extend our method to also allow the decomposition of fused structure-sparsity plus low rankness, providing a powerful tool for models with temporal information. Specifically, we develop a nontrivial modified alternating direction method of multipliers with at least local convergence. Finally, we use both synthetic and real data to validate the excellence of our method. In the application of reconstructing two-stage cancer networks, “the Warburg effect” can be revealed directly.

## 1. Introduction

Learning a graphical model from high-dimensional but partial observations is ill posed, leading to infinitely numerous solutions. A possible approach to address this underdetermined problem is to impose a low complexity solution with a low-dimensional structure (geometry), such as the sparse vector [1], the low-rank matrix [2], and their combinations (the sparse and low-rank decomposition) [3].

One feasible way to learn such a graphical model is to capture any conditional independence between each pair of the variables with a sparsity prior. Under the assumption of multivariate normal distribution, this reconstruction can be simplified as an inverse of the covariance matrix through a penalized optimization plus a sparsity-induced regularization (Gaussian graphical model) as [4]

(1) $$\begin{array}{c} \hat{\Omega }=\underset{\Omega ⪰0}{\arg\;\min}\;Tr\left(\;S\Omega \;\right)-\log\det\Omega +\lambda {\left|\;\Omega \;\right|}_{1}, \end{array}$$

where *S* is the covariance matrix of the data, *Ω* = *S* ^−1^ represents its inverse, and *λ* denotes the tuning parameter for the sparsity-induced regularization.

**Figure 1:**
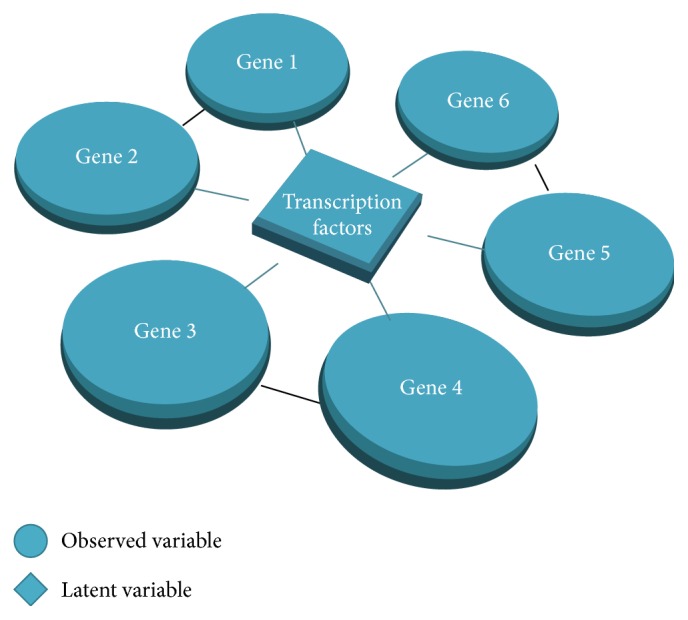
A network with a few latent variables. The edges between a pair of nodes represent that the two nodes are independent condition on remaining network.

Unfortunately, there is the possibility that a few of the variables are hidden or unobserved, thus requiring a latent model. Imagine a complex network with a few latent variables, each densely interacting with multiple observed variables. Thus, the sparsity assumption will not hold because of this latent structure (Figure 1). For instance, transcriptional factors (proteins) which regulate RNA transcriptions are not directly observed from whole-genome expressions (genechip or microarray). Therefore, an additive regularization (sparse plus low-rank recovery) has been developed to decompose the sparse interactions among the observed variables (sparsity) from a few latent variables (low rankness), that is, latent variables Gaussian graphical model (LVGGM) [5] as

(2) Ω^X,L^=arg minΩX−L≻0, L⪰0 ⁡TrSXΩX−L−log⁡det⁡ΩX−L+λ1ΩX1+λ2L∗,

where *S*_*X*_ is the covariance matrix of the observed data, *Ω*_*X*_ is the inverse of *S*, and *Ω*_*X*_ − *L* is the surl component of *Ω*_*X*_, corresponding to the latent variables *L* with low rank. *λ*_1_ and *λ*_2_ are the tuning parameters for sparsity and low rankness, respectively. The details of (1) and (2) will be depicted in Section 2.

An ultimate approach for such a sparse and low-rank recovery would be *ℓ*_0_ (base pursuit) plus the selection of exact ranks and thus a NP-hard problem [6]. A common relaxation is the use of *ℓ*_1_ plus the nuclear norm. An alternative relaxation is the use of concave penalties (Table 1, such as MCP, log or exponential type penalty, and SCAD), which have been verified to be very effective including biomolecular network reconstruction and image denoising [7, 8]. However, this concave approach so far has only been applied in sparse estimation problems.

The overall goal of this paper is therefore to develop a computational framework for a concave regularization with additive sparse and low-rank constraints [9] because of our desire to encode latent variables in a Gaussian graphical model but with insufficient samples, a very important issue in gene interaction network with latent regulatory factors. Below we want to justify why we chose a concave approach.

We chose an additive concave regularization because it does not require the strong irrepresentable condition, particularly when sample complexities are not sufficient [10]. This irrepresentable condition is highly relevant for biological observations (*p* ≫ *n* and often with very limited *n*). In contrast, a strong irrepresentable condition is necessary for Lasso in order for it to be selection consistent (requiring more sample complexities).

We chose a concave regularization also because of its parameter-estimation consistency, able to correct the intrinsic estimation biases from Lasso [11]. Here the bias is defined as the inevitable shrinkage introduced by Lasso, linearly expanded along with the tuning parameter *λ*. This bias issue has been noted during a regression setting [12] and a sparse precision matrix estimation [7].

Finally, we chose a concave regularization because an error bound has already been established for sparse least square problems [10]. C.-H. Zhang and T. Zhang established a bound by imposing an appropriate *ℓ*_2_ regularity condition such that a family of column-normalized matrices can guarantee a desirable estimation under an appropriate sparsity assumption [10], leading to the error bound that is no worse than Lasso. This general result holds for the entire concave regularization family including the bridge penalties (*ℓ*_*q*_, *q* < 1). Note that both of our regularizations belong to the family of bridge penalties.

As aforementioned we have intentionally selected a regularization scheme from the general concave regularization family because of its established theoretical supremacy (improve the variable selection accuracy and gain the oracle properties by reducing the bias of Lasso) [12, 13]. Our motivation is to provide the community with at least a computational alternative when some additive concave penalties are critically needed for some niches of timely applications (really demanding the oracle property), particularly when observations are limited (such as gene expression arrays).

Overall, the first contribution of our effort is having provided a novel bridge-nuclear penalty to induce a low-rank structure as well as a bridge-fused penalty to induce a fused-structural sparsity [14]. Note that we have explicitly derived the proximity operators [15] for these penalties (concave), respectively, an essential and important step towards a gradient-based optimization [16]. The structural sparsity here is used to join multiple graphical models to compare their differences (evolution of network structures, different stages and types of tumorigenesis, and other network comparison problems).

Our second contribution is to provide a modified alternative direction multiplier method (modified ADMM) [17] to numerically optimize these concave estimators (via their proximity operators), leading to at least some local solutions. We chose ADMM as the optimization method because we can prove its local convergence. We note the convergence of ADMM when applied to convex LVGGM as has been proved by [18].

Our third contribution is having provided a vigorous proof for the local convergence of this ADMM based on a framework for analyzing linear constrained optimization algorithms [19]. We use variational inequality to derive the contraction property in each iteration, which guarantees the monotonic convergence to a stationary point. Our experiments using both synthetic and real data indicated better performances compared to the classical convex regularizations. Overall we have developed an unified computational approach for additive concave regularization.

## 2. Latent Gaussian Graphical Model with Additive Concave Regularization 

### 2.1. Notation

We defined our notations as follows. For *n*-dimensional vector *x* ∈ *ℛ* ^*n*^, for *q* > 0, we define the *ℓ*_*q*_ norm ‖*x*‖_*q*_ = (∑_*i*=1_^*n*^|*x*_*i*_|^*q*^)^1/*q*^. Here we note the *ℓ*_*q*_ norm is a quasinorm for 0 < *q* < 1. *I*_*p*_ ∈ *ℛ* ^*p*×*p*^ represents the identity matrix. For rectangular matrices *M*, *N* in *ℛ* ^*p*×*q*^, the spectral norm ‖*M*‖ is the largest singular value, ‖*M*‖ = sup_*x*∈*ℛ*^*q*^_⁡(‖*Mx*‖/‖*x*‖), and the nuclear norm ‖*M*‖_*∗*_ is the sum of the singular values. The Frobenius norm ‖*M*‖_*F*_ is the *l*_2_ norm of the singular values; ‖*M*‖_*F*_ = (Tr⁡(*M* ^*T*^ *M*))^1/2^. The *l*_*∞*_ norm is defined by ‖*M*‖_*∞*_ = max_*i*,*j*_⁡*M*_*ij*_. The Hadamard product of *M*∘*N* is defined as [*M*∘*N*]_*ij*_ = [*M*]_*ij*_[*N*]_*ij*_. The function sign(*x*) extracts the sign of a real number *x*.

### 2.2. Gaussian Graphical Model with Concave Regularization

In this section, we briefly review the related works on Gaussian graphical model (GGM) and latent variable Gaussian graphical model (LVGGM).

A GGM also known as a Gaussian-Markov random field-based method is defined with respect to a graph *G*(*V*, *E*). The set of nodes *V* consists of *p* individual variables (features) with *n* observations *X* = (*x*_1_, *x*_2_,…, *x*_*p*_)′ ∈ *ℛ* ^*p*×*n*^ under the multivariate normal distribution *𝒩*(*μ*, Σ). The edges represent the conditional independencies among the variables, where the edges *e*_*ij*_ ∉ *E*, if and only if *x*_*i*_ and *x*_*j*_ are independent, conditioned on the remaining variables. With the Gaussian assumption, this conditional independence for any pairs of nodes *x*_*i*_ and *x*_*j*_ is equivalent to their partial correlation *ρ*_*ij*_ being zero, so that $e_{ij}\notin E\Leftrightarrow \rho_{ij}=\Sigma_{ij}^{-1}/\sqrt{\Sigma_{ii}^{-1}\Sigma_{jj}^{-1}}=0.$ It is important to note that such an equivalence requires that the random variables *X* be sampled from the families of distributions characterized with a semigroup property, including multivariate Gaussian, elliptical, multivariate hypergeometric, multivariate negative hypergeometric, multinomial, and Dirichlet distributions [20].

Therefore, the GGM can be learned by estimating its precision matrix, the inverse of the covariance matrix, in which each off-diagonal element represents its conditional independence. This estimation can be formulated as a sparsity-regularized optimization problem (1) with the sparsity assumption about the network structure. Note that there are a number of alternatives to formulate this sparsity-induced regularization. For instances, convex regularizations with the group *l*_2_ norm have been used to estimate the precision matrix [4, 21]. These regularization schemes have been extensively discussed, whose bounds have been estimated and even analytically proved [22, 23].

A more recent effort to achieve better statistical properties, including the oracle properties and unbiased estimation, has been developed by Fan et al., using a folder concave-based regularization [7] as follows:

(3) Ω^=arg minΩ⪰0 ⁡TrSΩ−log⁡det⁡Ω+λ∑i=1p ∑j=1pSCADλ,aωij.

The SCAD is symmetric and a quadratic spline on [0, *∞*, whose first-order derivative is given by SCAD_*λ*,*a*_′(*x*) = *λ*{*I*(|*x*| ⩽ *λ*) + ((*aλ* − |*x*|)_+_/(*a* − 1)*λ*)*I*(|*x*| > *λ*)} with a suggested value of *a* = 3.7. Model (3) has some admirable performances in practice but still a few limitations. At least it is difficult to be extended to include possible complex and additive regularization schemes, such as the combination of sparsity with low rankness or structural sparsity. Still the most challenging is nonconvex nature for all concave-based approaches, possibly leading to many local solutions. Thus, one of the goals of this paper is to extend Fan et al.'s work to more complex and even additive regularization schemes.

### 2.3. Latent Gaussian Graphical Model with Additive Concave Regularization

A Gaussian graphical model can be incorporated with a few hidden variables (such as the latent Gaussian Graphical Model). Let *X* ∈ *ℛ* ^*p*^ be the observed variables and *Z* ∈ *ℛ* ^*r*^  (*r* ≪ *p*) the latent ones. Here typically we assume the number for those latent variables is small (low rankness). Thus, (*X*, *Z*) can be jointly sampled from a multivariate normal distribution. Suppose its covariance matrix is $\Sigma_{\left(X,Z\right)}=\left(\begin{array}{cc} \Sigma_{X} & \Sigma_{XZ}\\ \Sigma_{ZX} & \Sigma_{Z} \end{array}\right)$ and its corresponding precision matrix is $\Omega_{\left(X,Z\right)}=\left(\begin{array}{cc} \Omega_{X} & \Omega_{XZ}\\ \Omega_{ZX} & \Omega_{Z} \end{array}\right)$. Hence Σ_*X*_^−1^ = *Ω*_*X*_ − *Ω*_*XZ*_ *Ω*_*Z*_^−1^ *Ω*_*ZX*_ is called the Surl component of *Ω*_*X*_, where *Ω*_*X*_ is a sparse matrix and *Ω*_*XZ*_ *Ω*_*Z*_^−1^ *Ω*_*ZX*_ is denoted as *L*, being a low-rank matrix due to *r* ≪ *p*. Chandrasekaran introduced a regularized optimization with multiple additive terms, named as the latent variable graphical model (2) [5], and the error bound of LVGGM is proofed by Meng et al. [24]. Here *λ*_1_ and *λ*_2_ are two tuning parameters, which are often hard to choose. An empirical way is to use certain information criteria or conduct *K*-fold cross-validations.

We propose a novel bridge-nuclear penalty to induce a low-rank structure as follows.

**Definition 1 .**

For a matrix *M* ∈ *ℛ* ^*n*×*n*^, suppose its singular value decomposition is *M* = *U*Σ*V*′, with *U*, Σ, *V* ∈ *ℛ* ^*n*×*n*^ the 1/2 quasinuclear norm of *M* being defined as ‖*M*‖_*♣*_ = (∑_*i*=1_^*n*^(*σ*_*i*_)^1/2^)^2^.

By employing the 1/2 quasinorm (bridge penalty) plus the 1/2 quasinuclear norm (bridge-nuclear penalty), we formulate our latent Gaussian graphical model with additive concave penalties as

(4) Ω^X,L^=arg minΩX−L≻0, L⪰0 ⁡TrSΩX−L−log⁡det⁡ΩX−L+λ1ΩX1/21/2+λ2L♣1/2.

Note again, our concave LVGGM does not need strong irrepresentable condition and thus can be applied to low sample complexity.

### 2.4. Joint Multiple Latent Gaussian Graphical Model with Additive Concave Regularization

To demonstrate the applicability of our additive approach, we purposely include a fused-structural sparsity, being used together with the aforementioned sparsity and low rankness. Here a total of three penalties are additively combined, potentially useful to model a network comparison problem with latent variables (joint latent variable Gaussian Graphical Model, JLVGGM). The evolution of biological and some other networks (such as social network) often has some invariant portion over the progression, which can thus be captured by our fused regularization over *k* individual snapshots. We consider this a potentially very useful approach to model a regulatory network in biology, since many gene interactions will remain invariantly imposed by their functional constraints (house-keeping, etc.).

The most commonly used constraint so far assumes that network evolution is gradual and local, representing mainly sporadic and minor structural changes, with most of the systems remaining intact. For instance, Guo et al. developed a joint Gaussian graphical model to learn multiple snapshots, assuming their biological networks being only partially changed [25]. Recently, Danaher et al. [26] used a fused-lasso scheme to model multiple stages of tumorigenesis.

As an extension to Danaher's fused graphical model with latent variables, also as an example to demonstrate our additive strategy with a structural sparsity, we formulate a joint model with latent variables. Suppose *X*_1_ ∈ *ℛ* ^*p*×*n*_1_^, *X*_2_ ∈ *ℛ* ^*p*×*n*_2_^,…, *X*_*k*_ ∈ *ℛ* ^*p*×*n*_*k*_^ are independent and identically distributed from *𝒩*(*μ*_*k*_, Σ_*k*_). We formulate our joint model with latent variables as

(5) Ω^Xi,L^i=arg minΩXi−Li≻0, Li⪰0⁡ ∑i=1kTrSiΩXi−Li−log⁡det⁡ΩXi−Li+∑i=1kλ1iΩXi1/21/2+λ2iLi♣1/2+∑i<j′k−1λ3iΩXi−ΩXj1/21/2,

where *λ*_3*i*_, *i* = 1,2,…, *k* − 1 represent the similarity measure among the temporal networks according to the tuning parameters.

## 3. Modified Alternating Direction Method of Multipliers

In this section, we want to establish the algorithm and its convergence of the modified alternating direction method of multipliers (ADMM). We applied this numerical method to our graphical model with latent variables. First we derived the proximity operators individually for *ℓ*_1/2_, the 1/2 quasinuclear norm ‖·‖_*♣*_ (bridge-nuclear penalty), and the fused *ℓ*_1/2_. With these proximity operators, we design a gradient-based but nonsmooth optimization based on alternating direction method of multipliers.

### 3.1. Proximity Operator

**Theorem 2 .**

The proximity operator of ‖*x*‖_1/2_^1/2^ is the global solution for the following problem:

(6) $$\begin{array}{c} \underset{\lambda {\left\|x\right\|}_{1/2}^{1/2}}{\mathrm{Prox}}\left(\;y\;\right)=\underset{x}{\arg\;\min}\left\{\;\frac{1}{2}{\left\|\;x-y\;\right\|}_{2}^{2}+\lambda {\left\|\;x\;\right\|}_{1/2}^{1/2}\;\right\}. \end{array}$$

One has

(7) $$\begin{array}{c} \underset{\lambda {\left\|x\right\|}_{1/2}^{1/2}}{\mathrm{Prox}}\left(\;y\;\right)=\left\{\begin{array}{cc} \frac{2}{3}y\left(1+\cos\left(\frac{2\pi }{3}-\frac{2}{3}\arccos\left(\frac{\lambda }{4}{\left(\frac{\left|y\right|}{3}\right)}^{-3/2}\right)\right)\right), & y>\frac{3}{2}\sqrt[{3}]{\lambda^{2}}\\ 0, & \left|y\right|⩽\frac{3}{2}\sqrt[{3}]{\lambda^{2}}\\ \frac{2}{3}y\left(1+\cos\left(\frac{2\pi }{3}-\frac{2}{3}\arccos\left(\frac{\lambda }{4}{\left(\frac{\left|y\right|}{3}\right)}^{-3/2}\right)\right)\right), & y<-\frac{3}{2}\sqrt[{3}]{\lambda^{2}}. \end{array}\right. \end{array}$$

**Proof**

Since the subdifferential of a nonconvex function is not well defined, we resolve the optimization problem (6) as follows.

If Prox_*λ*‖*x*‖_1/2_^1/2^_(*y*) = 0 and *x* > 0, it implies that $(1/2)x^{2}-xy+\lambda \sqrt{x}⩾0$, which yields $y⩽x/2+\lambda /\sqrt{x}⩽(3/2)\sqrt[{3}]{\lambda^{2}}$. By symmetry, we have $y⩾-(3/2)\sqrt[{3}]{\lambda^{2}}$ in the case Prox_*λ*‖*x*‖_1/2_^1/2^_(*y*) = 0 and *x* < 0.

Otherwise, when Prox_*λ*‖*x*‖_1/2_^1/2^_(*y*) ≠ 0, the global solution of (6) is one of the three roots for the following algebraic equation: $(1/\sqrt{\left|x\right|})({\sqrt{\left|x\right|}}^{3}-y\sqrt{\left|x\right|}+\lambda (sign\left(x\right)/2))=0$, which is derived by taking the derivative on both sides of (6). With similar calculations like [27], the equation

(8) $$\begin{array}{c} {\sqrt{\left|x\right|}}^{3}-y\sqrt{\left|x\right|}+\lambda \frac{sign\left(x\right)}{2}=0 \end{array}$$

has three roots in a compact trigonometric form as

(9) x=2y3cos⁡π+2kπ−arccos⁡λ/4y/3−3/23,k=0,1,2,

under the conditions *x* > 0 and $y>(3/2)\sqrt[{3}]{\lambda^{2}/2}$. It is validated that (6) will reach a global minimum, when *k* = 0. Since *x* and *y* are either positive or negative simultaneously, we have

(10) x=2−y3cos⁡π−arccos⁡λ/4−y/3−3/23,y>32λ223.

Similarly we have

(11) x=2−y3cos⁡π−arccos⁡λ/4−y/3−3/23,y<−32λ223.

By taking a square root on both sides of (10) and (11) within the domain $\left[\left(-\infty ,-\left(3/2\right)\sqrt[{3}]{\lambda^{2}}\right)\cap (-\infty ,-(3/2)\sqrt[{3}]{\lambda^{2}/2})]\cup [((3/2)\sqrt[{3}]{\lambda^{2}},+\infty )\cap ((3/2)\sqrt[{3}]{\lambda^{2}/2},+\infty )\right]$, the statement in Theorem 2 is proven.

**Theorem 3 .**

Assuming *X*, *Y* ∈ *ℛ* ^*n*×*n*^, the proximity operator of the 1/2 quasinuclear norm is given as the global minimum of

(12) $$\begin{array}{c} \underset{\lambda {\left\|X\right\|}_{♣}^{1/2}}{\mathrm{Prox}}\left(\;Y\;\right)=\underset{X}{\arg\;\min}\left\{\;\frac{1}{2}{\left\|\;X-Y\;\right\|}_{F}^{2}+\lambda {\left\|\;X\;\right\|}_{♣}^{1/2}\;\right\}, \end{array}$$

where *d*(*X*), *d*(*Y*) ∈ *ℛ* ^*n*×1^ represent the single value of *X* and *Y*, respectively, in nonincreasing order. *U*_*Y*_ and *V*_*Y*_ are the left and right orthogonal matrices with the singular value decomposition of *Y* = *U*_*Y*_Diag(*d*(*Y*))*V*_*Y*_′. We have

(13) $$\begin{array}{c} \underset{\lambda {\left\|X\right\|}_{♣}^{1/2}}{\mathrm{Prox}}\left(\;Y\;\right)=U_{Y}Diag\left(\;\left[\;t_{1},t_{2},\ldots ,t_{n}\;\right]\;\right)V_{Y}^{\prime }, \end{array}$$

with *t*_*i*_ = (2/3)*σ*_*i*_(1 + cos⁡(2*π*/3 − (2/3)arccos⁡((*λ*/4)(|*σ*_*i*_|/3)^−3/2^))), if $\sigma_{i}>(3/2)\sqrt[{3}]{\lambda^{2}}$ and *t*_*i*_ = 0, for $\sigma_{i}⩽(3/2)\sqrt[{3}]{\lambda^{2}}$ else.

**Proof**

Through von Neumann's trace inequality [28], we have

(14) X−YF2TrX−YX−Y′=TrXX′−2 TrXY′+TrYY′=∑i=1ndi2X−2 TrXY′+∑i=1ndi2Y⩾∑i=1ndi2X−2diXdiY+di2Y.

The equality holds if and only if *Y* = *U*_*Y*_Diag(*d*(*X*))*V*_*Y*_^*T*^. Then the optimization is reduced to

(15) arg mindiX>0 ⁡12diX2−diXdiY+12diY2+λdiX.

We note here (15) is just a special case of (6). This completes the proof of Theorem 3. We note this regularization would induce a low-rank approximation of *Y* due to the threshold of *t*_*i*_.

**Theorem 4 .**

The proximity operator of fused *ℓ*_1/2_ regularization is given as the global minimum of

(16) ProxλXij2−Xij11/21/2⁡Aij1,Aij2=arg minXij1,Xij2 ⁡12∑k=1,2Xijk−AijkF2+λXij2−Xij11/21/2.

One has

(17) $$\begin{array}{c} \underset{\lambda {\left\|X_{\mathrm{ij}}^{\left(2\right)}-X_{\mathrm{ij}}^{\left(1\right)}\right\|}_{1/2}^{1/2}}{\mathrm{Prox}}\left(\;A_{ij}^{\left(1\right)},A_{ij}^{\left(2\right)}\;\right)=\left\{\begin{array}{cc} \left(\frac{A_{ij}^{\left(1\right)}+A_{ij}^{\left(2\right)}-K_{ij}}{2},\frac{A_{ij}^{\left(1\right)}+A_{ij}^{\left(2\right)}+K_{ij}}{2}\right), & A_{ij}^{\left(1\right)}-A_{ij}^{\left(2\right)}>\frac{3}{2}\sqrt[{3}]{\lambda^{2}},\\ \left(\frac{A_{ij}^{\left(1\right)}+A_{ij}^{\left(2\right)}}{2},\frac{A_{ij}^{\left(1\right)}+A_{ij}^{\left(2\right)}}{2}\right), & \left|A_{ij}^{\left(1\right)}-A_{ij}^{\left(2\right)}\right|⩽\frac{3}{2}\sqrt[{3}]{\lambda^{2}},\\ \left(\frac{A_{ij}^{\left(1\right)}+A_{ij}^{\left(2\right)}+K_{ij}}{2},\frac{A_{ij}^{\left(1\right)}+A_{ij}^{\left(2\right)}-K_{ij}}{2}\right), & A_{ij}^{\left(1\right)}-A_{ij}^{\left(2\right)}<-\frac{3}{2}\sqrt[{3}]{\lambda^{2}}, \end{array}\right. \end{array}$$

with *K*_*ij*_ = (2/3)(*A*_*ij*_^(1)^ − *A*_*ij*_^(2)^)(1 + cos(2*π*/3 − (2/3)arccos⁡((*λ*/4)(|*A*_*ij*_^(1)^ − *A*_*ij*_^(2)^|/3)^−3/2^))).

**Proof**

If *X*_*ij*_^(1)^ = *X*_*ij*_^(2)^, this yields *X*_*ij*_^(1)^ = *X*_*ij*_^(2)^ = (*A*_*ij*_^(1)^ + *A*_*ij*_^(2)^)/2. If *X*_*ij*_^(1)^ < *X*_*ij*_^(2)^; by taking derivation, we have

(18) Xij1−Aij1−λsignXij2−Xij12Xij2−Xij1=0,Xij2−Aij2+λsignXij2−Xij12Xij2−Xij1=0.

Denoting *X*_*ij*_^(2)^ − *X*_*ij*_^(1)^ = *K*_*ij*_ > 0, we have

(19) $$\begin{array}{c} K_{ij}+\left(\;A_{ij}^{\left(1\right)}-A_{ij}^{\left(2\right)}\;\right)+\frac{\lambda \;sign\left(K_{ij}\right)}{\sqrt{\left|K_{ij}\right|}}=0. \end{array}$$

We note here that (19) is just the same form as (8).

Therefore, we have *X*_*ij*_^(2)^ = (*A*_*ij*_^(1)^ + *A*_*ij*_^(2)^)/2 + *K*_*ij*_/2 and *X*_*ij*_^(1)^ = (*A*_*ij*_^(1)^ + *A*_*ij*_^(2)^)/2 − *K*_*ij*_/2. By taking a similar calculate when *X*_*ij*_^(1)^ > *X*_*ij*_^(2)^, we obtain our proposition.

## 4. Modified Alternating Direction Method of Multipliers

In this section, we provide an algorithm to approach the local solution of additive concave regularization problem (2). The Lagrangian of (2) is

(20) L=TrSX−log⁡det⁡X−Y,ΩX−L−X+λ1ΩX1/21/2+λ2L♣1/2+ρ2ΩX−L−XF2,

where *Y* ∈ *ℛ* ^*p*×*p*^ represents the corresponding dual variable. We propose a modified ADMM discretization to optimize the Lagrange as Algorithm 5 with at least local convergence.

Before we prove the convergence result, we need to prove the following contraction property which is the key for the proof of the convergence of general ADMM [29].

**Algorithm 5 (modified ADMM).**

 (1) Initialize *X*, *Ω*_*X*_, *L*, *Y* parameters *ρ*, *λ*_1_, *λ*_2_, *α*_1_ = 1
 for *i* = 1,2,…, *k* until convergence
 (2) *ω* ^(*k*)^ = (*X* ^(*k*)^, *Ω*_*X*_^(*k*)^, *L* ^(*k*)^, *Y* ^(*k*)^)^*T*^
 (3)

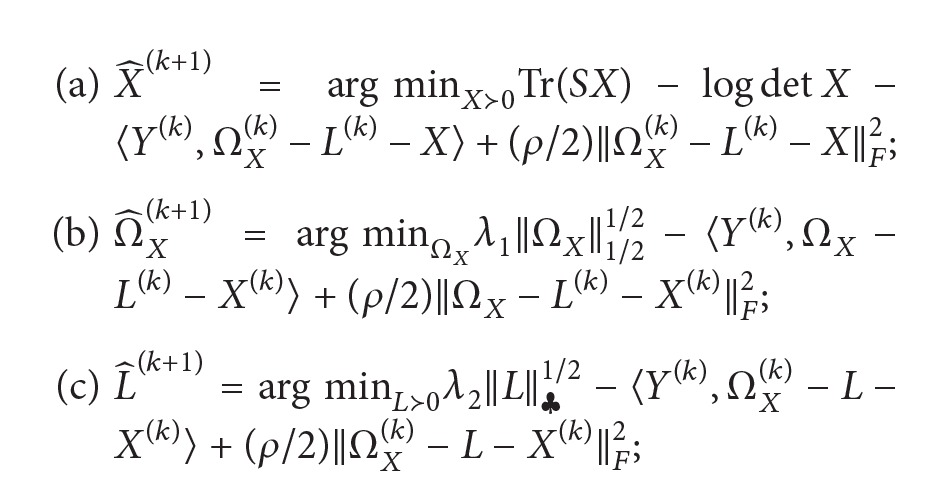

 (4) ${\hat{Y}}^{\left(k+1\right)}=Y^{\left(k\right)}-\rho \left({\hat{\Omega }}_{X}^{\left(k+1\right)}-{\hat{L}}^{\left(k+1\right)}-{\hat{X}}^{\left(k+1\right)}\right)$, $\hat{\omega }={\left({\hat{X}}^{\left(k+1\right)},{\hat{\Omega }}_{X}^{\left(k+1\right)},{\hat{L}}^{\left(k+1\right)},{\hat{Y}}^{\left(k+1\right)}\right)}^{T}$
 (5) $\alpha_{k+1}=\left(1+\sqrt{1+4\alpha_{k}^{2}}\right)/2$ and *γ* = (*α*_*k*_ − 1)/*α*_*k*+1_.
 (6) $\omega^{\left(k+1\right)}=\omega^{\left(k\right)}-\gamma (\omega^{\left(k\right)}-\hat{\omega })$.
 end

**Theorem 6 .**

Assume that *ω* ^*∗*^ = (*X* ^*∗*^, *Ω*_*X*_^*∗*^, *L* ^*∗*^, *Y* ^*∗*^)^*T*^ is a global optimal solution of (2) and *ω* ^(*k*)^ = (*X* ^(*k*)^, *Ω*_*X*_^(*k*)^, *L* ^(*k*)^, *Y* ^(*k*)^)^*T*^ is optimized by Algorithm 5. Thus the contradicted property holds; that is,

(21) ωk+1−ω∗F2⩽ωk−ω∗F2−γ−2+3γωk−ωk+1F2.

**Proof**

For any *X*, *Ω*_*X*_, *L*, *Y* ∈ *ℛ* ^*p*×*p*^, the optimality conditions (20) are

(22) Tr⁡SX−Tr⁡SX^k+1−log⁡det⁡X+log⁡det⁡X^k+1−Yk,ΩXk−Lk−X+Yk,ΩXk−Lk−X^k+1−ρX−X^k+1,Ωk−Lk−X^k+1⩾0,λ1ΩX1/21/2−λ1Ω^Xk+11/21/2−Yk,ΩX−Lk−Xk+Yk,Ω^Xk+1−Lk−Xk+ρΩX−Ω^Xk+1,Ω^Xk+1−Lk−Xk⩾0,λ1L1/21/2−λ1L^k+11/21/2−Yk,ΩXk−L−Xk+Yk,ΩXk−L^k+1−Xk−ρL−L^k+1,Ωk−L^k+1−Xk⩾0.

By denoting $T=\rho ({\hat{\Omega }}^{\left(k+1\right)}-\Omega^{\left(k\right)})-\rho ({\hat{L}}^{\left(k+1\right)}-L^{\left(k\right)})-\rho ({\hat{X}}^{\left(k+1\right)}-X^{\left(k\right)})$,

(23) Fω^=Y^k+1−Y^k+1Y^k+1ρ2Ω^Xk+1−X^k+1−L^k+1,O1=ρX^k+1−XkρΩ^Xk+1−ΩkρL^k+1−LkρY^k+1−Yk+−TT−T0,

we have

(24) TrSX−TrSX^k+1+λ1Ω1/21/2−λ1Ω^Xk+11/21/2+λ2L1/21/2−λ2L^k+11/21/2+ω−ω^,Fω^+O1⩾0.

Without loss of generality, we let *ω* = (*X*, *Ω*_*X*_, *L*, *Y*)^*T*^ = *ω* ^*∗*^, which yields $\rho \langle \hat{\omega }-\omega^{*},\omega^{\left(k\right)}-\hat{\omega }\rangle ⩾\langle \omega^{*}-\hat{\omega },F(\hat{\omega })+{𝒪}_{1}\rangle$. Using the optimal conditional (variational inequalities) [30], it follows that

(25) $$\begin{array}{c} \langle \;\omega^{*}-\hat{\omega },F\left(\;\omega^{*}\;\right)\;\rangle ⩾0. \end{array}$$

Since *F*(*ω*) is monotone, we have $\langle \omega^{*}-\hat{\omega },F(\hat{\omega })\rangle ⩾\langle \omega^{*}-\hat{\omega },F(\omega^{*})\rangle .$ Therefore, we have $\rho \langle \omega^{\left(k\right)}-\omega^{*},\omega^{\left(k\right)}-\hat{\omega }\rangle ⩾\rho {\left\|\hat{\omega }-\omega^{\left(k\right)}\right\|}_{F}^{2}+\delta$, with $\delta =\langle Y^{\left(k\right)}-{\hat{Y}}^{\left(k+1\right)},\rho (X^{\left(k\right)}-{\hat{X}}^{\left(k+1\right)})+\rho (L^{\left(k\right)}-{\hat{L}}^{\left(k+1\right)})-\rho (\Omega_{X}^{\left(k\right)}-{\hat{\Omega }}_{X}^{\left(k+1\right)})\rangle$. We denote

(26) $$\begin{array}{c} H=\left(\begin{array}{cccc} I_{p} & 0 & 0 & \frac{1}{2}I_{p}\\ 0 & -I_{p} & 0 & \frac{1}{2}I_{p}\\ 0 & 0 & I_{p} & \frac{1}{2}I_{p}\\ \frac{1}{2}I_{p} & \frac{1}{2}I_{p} & \frac{1}{2}I_{p} & \frac{1}{2}I_{p} \end{array}\right); \end{array}$$

thus the smallest eigenvalue is $\left(2-\sqrt{3}\right)/2$. We obtain

(27) ρω^−ωkF2+δ=ρXk−Xk+1ΩXk−ΩXk+1Lk−Lk+1Yk−Yk+1,HXk−Xk+1ΩXk−ΩXk+1Lk−Lk+1Yk−Yk+1=Trωk−ω^T·Hωk−ω^⩾2−32ρωk−ω^F2.

In summary, we have

(28) ω^k+1−ω∗F2ωk−γωk−ω^−ω∗F2=ωk−ω∗F2+γ2ωk−ω^F2−2γωk−ω∗,ωk−ω^⩽ωk−ω∗F2−γγ−2+3ωk−ω^F2⩽ωk−ω∗F2−γ−2+3γωk−ωk+1F2.

**Theorem 7 .**

The sequence *ω* ^(*k*+1)^ produced by Algorithm 5 from a given initial value converges monotonically to an optimal solution to problem.

**Proof**

From Theorem 6, we can easily get the following:

(1) ‖*ω* ^(*k*)^ − *ω* ^(*k*+1)^‖_*F*_ → 0.
(2) {*ω* ^(*k*)^} lies in a compact region.
(3) ‖*ω* ^(*k*)^ − *ω* ^*∗*^‖_*F*_ is monotonically nonincreasing and thus converging.

It follows that *ω* ^(*k*+1)^ is a Cauchy sequence and thus has a limit point $\overset{-}{\omega }$. Therefore we have *ω* ^(*k*+1)^ converging monotonically to $\overset{-}{\omega }$.

## 5. Numerical Evaluation

In this paper, we focus on the effective local convergence of ill-posed graphical model problems with additive concave regularization. The specific aim is to develop an additive regularization combining both sparsity and low rankness, essential for our latent graphical model. Again we chose a concave approach (instead of the popular convex methods) because of its presumptive advantage (the oracle property) especially when the data complexity is not sufficient (*p* ≫ *n*). It is thus important to first develop a robust numerical method to at least obtain a consistent local solution. We believe our methods are highly applicable to situations where the number of variables far exceeds their observations (large *p* small *n* problem, typical for most biological observations).

Below we use both artificial and real data to establish the effectiveness of our methods. We demonstrate that our nonconvex regularization can reduce the biases, as illustrated in its applications to the selected biological and financial problems. In addition, we assess the performance of our concave models comparing with the corresponding convex ones.

### 5.1. Artificial Data

Our concave graphical model is supposed to perform better as an asymptotically unbiased estimator. We generate our artificial data according to the following steps.

We generate 5% nonzero entries uniformly from a 50 × 50 matrix $\hat{\Omega }$. Each nonzero element in this ${\hat{\Omega }}_{ij}$ is sampled from a Gaussian distribution of *𝒩*(0, *σ* ^2^). We construct a symmetric precision matrix as $\Omega =\hat{\Omega }+{\hat{\Omega }}^{T}$. To guarantee the positive definiteness of this *Ω*, we update the matrix *Ω* = *Ω* + 1.1*I*_50_ iteratively, such that all of its eigenvalues be greater than zero. We randomly choose 45 columns and their corresponding rows as a subblock matrix *Ω*_*O*_ form *Ω*; thus the remaining 10% variables are latent. Finally, we take the inverse of the precision matrix Σ_*O*_ = *Ω*_*O*_^−1^ as its original covariance matrix to sample a Gaussian distribution.

We divide our experiments into two groups with the increasing sample complexity: *p* = (1/2)*N*, *N* random samples from the multivariate Gaussian distribution *𝒩*(0, *σ* ^2^), with *σ* ^2^ = 3. Important to note is that in order to test the effectiveness of our method for an ill-posed problem (*p* ≫ *n*), we purposely only increase the number of observations while keeping their variances a constant.

We perform the following comparisons:

1. convex Gaussian graphical model with latent variables (LVGGM) [5];
2. concave Gaussian graphical model with latent variables (concave LVGGM).

**Figure 2:**
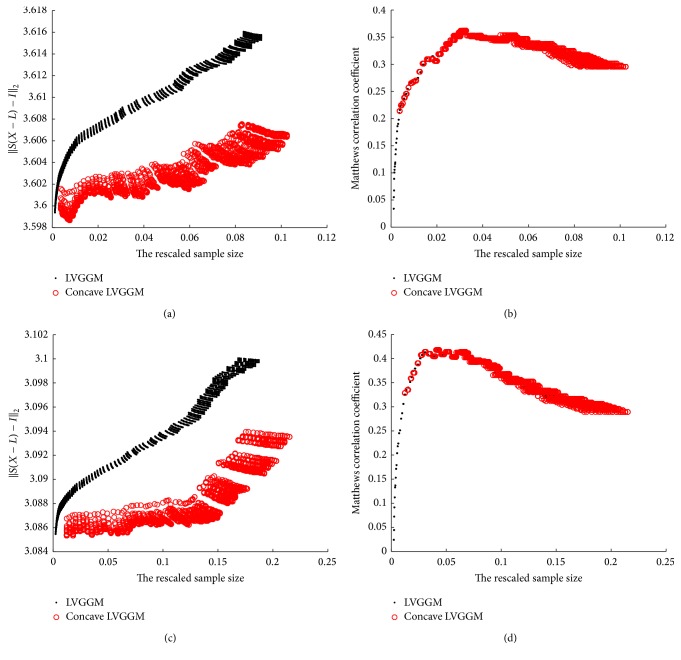
Simulation chains for the graphical model with latent variables. The estimation error ‖*S*(*X* − *L*) − *I*‖_2_ versus the rescaled sample size *n*/(*s*log⁡(*p*) + *r*log⁡(*p*)) ((a) and (c)), where *s* is the number of nonzero entries of *X* and *r* is the rank of *L*. The MCC rate versus the rescaled sample size ((b) and (d)). We note the sample complexity is *n* = (1/2)*p* for (a) and (b) and *n* = *p* for (c) and (d).

We use this example to validate that our method has better performance than the classic LVGGM. Firstly, for parameters estimation consistence as Figure 2 shows, our concave LVGGM estimate parameters more accurately than the classic model in [5], even with a local solution. We want to note that our method performs better with the larger observed data in Figure 2(c) but is significantly superior to the classical method with inadequate data in Figure 2(a). This is very important with biological application usually with very insufficient observation.

Finally, to assess the consistence of model selection for our methods, we use the Matthews correlation coefficient (MCC ∈ [−1,1]) to quantify recovery merits, where

(29) MCC=TP×TN−FP×FNTP+FPTP+FNTN+FPTN+FN

with TP, TN, FP, and FN being the numbers of true positives, true negatives, false positives, and false negatives. The larger the MCC is, the better the reconstruction model performs. Figures 2(b) and 2(d), depicting LVGGM and concave LVGGM, respectively, are both model selection consistent.

### 5.2. The Biomolecular Network of Medulloblastoma

To demonstrate that our method performs consistent in both artificial data and real application, we apply our method to human medulloblastoma. Medulloblastoma is the most common form of childhood brain tumors. This cancer has at least four subgroups, including the WNT subgroups and the sonic hedgehog (SHH), plus the subgroups 3 and 4, with the molecular etiology remaining elusive for the latter two. In China, patients with medulloblastomas are still largely treated with universal and aggressive procedures, combining radical surgery, radiation, and chemotherapy, which might in fact fail the subgroups 3 and 4 (poor prognosis with unknown reasons), or probably have overtreated the WNT subgroup (more differentiated). Thus, it is important to develop a methodology to clearly distinguish and identify the key molecular markers and their interactions at system level, representing each of the subgroups decisively. We hope our results will yield a set of genes in some given structures which can be used as the signatures (biomarkers) to guide diagnosis, treatment, and prognosis in future.

**Figure 3:**
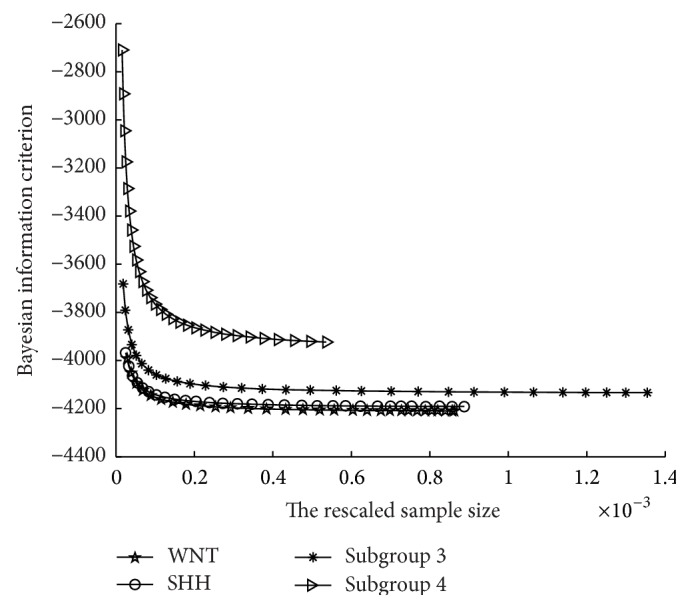
BIC scores. The BIC score for our concave LVGGM over the rescaled sample size *n*/(*s* log(*p*) + *r* log(*p*)). We use a grid-based search for the local minimums for (*λ*_1_, *λ*_2_); the parameters are (*λ*_1_, *λ*_2_) = (0.0005,0.001), (0.00038966,0.001), (0.0002931,0.001), (0.00047241,0.001) for subgroups WNT, SHH, 3, and 4, respectively.

The gene expression data are publicly available from the National Center for Biotechnology Information (NCBI), consisting of 73 individual cancer samples (8, 10, 16, and 39 samples for WNT, SHH, subgroup 3, and subgroup 4, resp.), each labeled with a specific subgroup (accession number: GDS4296) [31]. Firstly, We select 1146 genes out of 54676, whose expressions show significantly larger variance across both of the cancer samples. In order to tune our parameters we use Bayesian information criterion (BIC):

(30) BIC=−2−log⁡det⁡X−L+TrSX−L+γklog⁡n

to select the tuning parameters *λ*_1_, *λ*_2_ with a constant parameter *γ*. Here *k* presents the nonzeros numbers of matrix *X*, while *n* denotes the total sample numbers. We use BIC because it is intrinsically incline to identify the “true” model while being asymptotically consistent in selecting such a model. We did consider another criterion such as Akaike information criterion (AIC) as it tends to explain the data, thus suffering the risk of overfitting. Since the BIC does not have a closed form with respect to *λ*_1_ and *λ*_2_, we carry out a grid-based screening (Figure 3).

**Figure 4:**
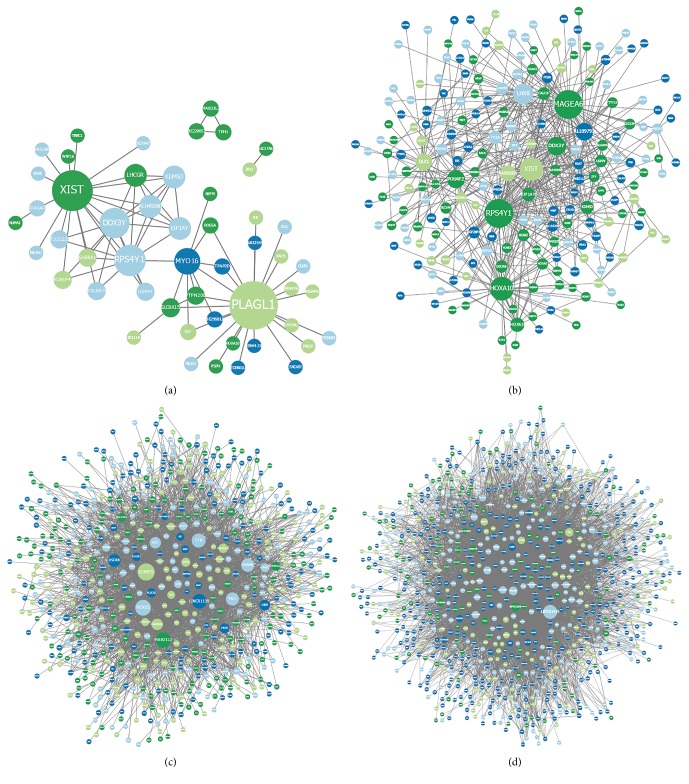
Topological for the biomolecular network of medulloblastomas. The sizes of nodes correspond to the degrees of their interactions. The colors represent similar classes according to the pairwise Pearson correlations of gene expressions. Demonstrated here is the absolute essentiality of using conditional independence to interpret the apparent correlations between gene expressions. (a) represents the WNT subgroup; (b) the SHH subgroup; (c) the subgroup 3; and (d) the subgroup 4. The regularization parameters are chosen to optimize BIC score.

As demonstrated in Figure 4(a), the famous WNT/beta-catenin pathway is recovered, including WNT16 and XIST. The WNT pathway is required for basic developmental processes, such as cell-fate specification, progenitor-cell proliferation, and the control of asymmetric cell division. Identified here is the canonical pathway, which inhibits the beta-catenin degradation complex. Our results thus strongly support the etiological role of the canonical WNT signaling in the pathogenesis of this subgroup of tumors [32]. As illustrated in Figure 4(b), part of the SHH signaling pathway is identified, including oncogene MAGEA, which is associated with shorter survival of tumor cells [33]. Since SHH subgroup medulloblastomas develops from cerebellar granule neuron progenitors, which are supposed to guide axon growth into muscles, it is our speculation that cells of this subgroup of tumors might interact with muscle cells sometime during early development. Interestingly, many modules identified here including TAC1 and TTR,are implicated in some sensory-related activities during early development for subgroup 3 (Figure 4(c)). Consistent with our observations, those sensory genes are overexpressed for this subgroup of tumors according to the independent studies [32]. It is clearly distinguishable from the other three, particularly when combined with the sensory basis (see above). However, note that the genes of subgroup 4 are more randomly distributed than the other three, without the apparent modular structures. Interestingly, a number of important oncogenes and tumor suppressor genes are found such as FOXG1 (Figure 4(d)).

### 5.3. The Structural Changes on Gene Regulatory Networks Occurring during the Progression of Human Lung Cancers

To indicate our concave additive regularizations can be used to model a structured sparsity (fused sparsity). We perform the following experiment to identify the structural changes on gene regulatory networks occurring during the progression of human lung cancers. The lung cancer dataset contains 22283 microarray-derived gene expression measurements from large airway epithelial cells sampled from 97 patients with lung cancer and 90 controls [34]. The data are publicly available from the Gene Expression Omnibus [35]; its accession number is GDS2771.

We first selected the 36 most important biological modules (CDK4, CDK6, CDK2AP2, BCL2, XIAP, BAX, CASP3, CASP8, CASP9, FOXA2, HNF4A, EBP, HGF, NFKB2, STAT3, IL6, IL10, HIF1AN, MYC, GSK3B, TP53, PTEN, RB1, MDM2, PDGFRA, SOX2, PIK3CA, FGFR1, IGF1R, EPHA2, MET, EGFR, DDR2, KEAP1, KRAS, and AKT1) out of the whole genome for lung cancer according to [36]. The goal of this experiment is to find out if our method can consistently detect key structural changes on the level of gene regulatory networks associated with lung cancer. It is well established that many protein-factors are directly or indirectly engaged in the control of transcripts. Also important to note is the availability of gene annotation information, which is still limited mainly for some of the key and frequently expressed genes. We take the notion that the network structure will largely determine the key aspects of biological functionality [37].

**Figure 5:**
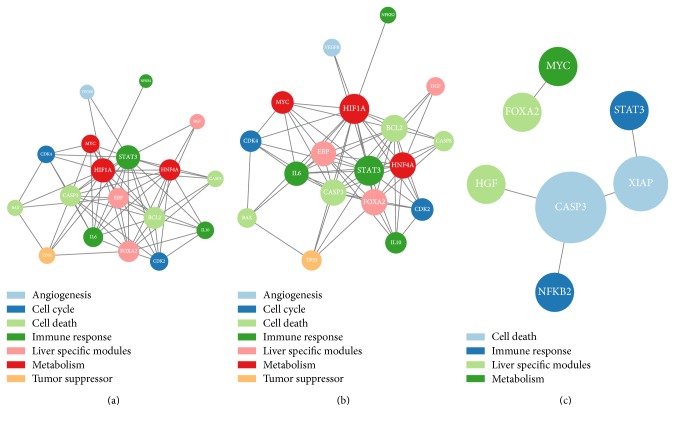
Gene regulatory networks occurring during the progression of human lung cancers. The network reconstructed in 75% confidence (the edges emerge at least 750 times) under 1000 times random permutations. The normal stage (a) and the cancer stage (b). Changes about edges between normal stage and cancer stage (c). The parameters are *λ*_11_ = 0.003, *λ*_21_ = 0.006, *λ*_31_ = 0.001, *λ*_12_ = 0.8*λ*_11_, and *λ*_21_ = *λ*_22_. The regularization parameters are manually chosen to induce high sparsity for better visualization and highlighting the dominating edges.

We want to know if there are any key differences between normal and cancer cells. We use the 36 key biological modules to reconstruct a two-stage gene regulatory network by our Concave JLVGGM. To approach statistical significance, we do a random permutation to the tuning parameter *λ*_11_ for 1000 times. According to the second law of thermodynamics, it implies that the cancer network becomes unstable during the progress of tumorigenesis, consistent with the notion that instability is a common phenotype of cancer cells. The tuning parameters are taken as *λ*_12_ = 0.8*λ*_11_. An interesting phenomenon we observed during our modeling effort is as follows: we found that the abnormal metabolism and apoptosis may be closely related to the progression of lung cancers according to Figure 5. As “the Warburg effect” suggests, most proliferation of cancer cells relies on aerobic glycolysis. Thus, the abnormal metabolism we detected in the cancer cells here is intriguing and significant. It is widely known that apoptosis plays a critical role in cancer and particularly lung cancer. However, based on our structural results (undirected graph), we do not know how these changes would be causally related.

## 6. Conclusion

We have developed a concave regularization approach for LVGGM for low sample complexity as well as for reducing the biases. Computational method based on proximity operators is provided with at least local convergence. Our methodology establishes its practical value as demonstrated by the numerical results (see Figure 2). Finally, as a future direction, we plan to assess a fully Bayesian interpretation of concave LVGGM. This may be helpful for an advisable choice of the tuning parameter as displayed in the framework of [38].

## Figures and Tables

**Table 1:** Examples of concave penalties *R*(*t*).

Penalty	*R*(*t*)	$\frac{d}{dt}R(t)$
*ℓ*_0_	*I*(|*t*| > 0)	None
Bridge (0 < α < 1)	|*t*|^α^	α|*t*|^α−1^ · sign⁡(*t*)
Capped-*ℓ*_1_	$\min\left(\frac{\gamma }{2},\left|t\right|\right)$	$I\left(\left|t\right|⩽\frac{\gamma }{2}\right)\cdot \mathrm{sign}\left(t\right)$
MCP	$\int_{0}^{\left|t\right|}{\left(1-\frac{x}{\gamma }\right)}_{+}dx$	${\left(1-\frac{\left|t\right|}{\gamma }\right)}_{+}\cdot \mathrm{sign}\left(t\right)$
SCAD	$\int_{0}^{\left|t\right|}1\wedge {\left(1-\frac{x-1}{\gamma -1}\right)}_{+}dx$	$1\wedge {\left(1-\frac{\left|t\right|-1}{\gamma -1}\right)}_{+}\cdot \mathrm{sign}\left(t\right)$
Log-type penalty	$\frac{1}{\log\left(\gamma +1\right)}\log\left(\gamma \left|t\right|+1\right)$	$\frac{1}{\log\left(\gamma +1\right)}\frac{\gamma }{\gamma \left|t\right|+1}$
Exponential-type penalty	$\frac{1}{1-\exp\left(-\gamma \right)}\left(1-\exp\left(-\gamma \left|t\right|\right)\right)$	$\frac{\gamma }{1-\exp\left(-\gamma \right)}\exp\left(-\gamma \left|t\right|\right)$

We note *I*(·) is a {0,1} value indicator function.
